# 903 Global Trends in Elderly Upper Extremity Burns

**DOI:** 10.1093/jbcr/iraf019.434

**Published:** 2025-04-01

**Authors:** Sara Ma, Carolyn Baldwin, Michael White, Mihaela-Elena Rapolti

**Affiliations:** Wayne State University School of Medicine; Wayne State University School of Medicine; Michael & Marian Ilitch Department of Surgery, Wayne State University School of Medicine, Detroit Medical Center; Wayne State University School of Medicine

## Abstract

**Introduction:**

Upper extremity (UE) burns are devastating for working adults, with the potential to significantly affect occupational capacity, family income, and activities of daily living. Elderly adults are even more likely to experience greater functional impairment and worsened outcomes due to co-morbidities and impaired healing. Major UE burns often require advanced reconstruction for nerve, skeletal, and soft tissue repair to preserve maximum functionality. However, institutions can vary in their ability to manage geriatric burn patients. The purpose of this study is to understand global trends in risk factors and clinical outcomes of geriatric UE burn injuries to guide resource allocation and burn prevention strategies.

**Methods:**

This study is a retrospective analysis of patient data from World Health Organization Global Burn Registry (WHO GBR). Patients 18+ years with reported burn injuries to shoulder, arm, hands, or wrists were grouped into non-geriatric (18-65 years) and geriatric (>65 years). Descriptive statistics and Student’s unpaired one-tailed T-tests were used to analyze the data. Significance was set at p=0.05.

**Results:**

WHO GBR contained 9277 entries from 20 countries from patients aged 0 to 95 years. There were 6773 UE burns to shoulder, axilla, arm, wrist and/or hand, of which 4257 were adults (age 18+) and nearly all (96.3%) from middle income countries. Non-geriatric adults (n = 3953) had sex distribution of 63% male and 37% female, while geriatric adults (n = 305) were more equal at 47% male and 53% female. Burn etiologies were similar in both groups, with flame being the most common. 50% of non-geriatric adults were discharged without physical impairment but associated with 30% mortality. Geriatric adults had worse outcomes, with a higher mortality (41%) and lower proportion of discharge home without physical impairment at 34%. Geriatric adults had higher discharge home with physical impairment (10%) and left more frequently against medical advice (12%) than non-geriatric adults (9% and 8%, respectively). Interestingly, non-geriatric literacy was associated with a significantly shorter stay (p = 0.02), while geriatric literacy was associated with a significantly longer stay (p = 0.01).

**Conclusions:**

Our study suggests that literacy status does not have the same effect on clinical outcomes in the elderly as in general adults. Perhaps the non-geriatric trend reflects increased perceived self-sufficiency associated with literacy, resulting in earlier discharge from hospital post-injury, while the geriatric trend suggests increased hypochondria or anxiety associated with literacy (i.e., health literacy) that compels longer hospitalization post-injury.

**Applicability of Research to Practice:**

Individual-level literacy-dependent material, such as inpatient education or burn prevention literature, may not be effective in elderly populations. Emphasis on system or community-level changes instead may improve elderly UE burn risk and outcomes.

**Funding for the Study:**

N/A

